# Do bisphosphonates cause femoral insufficiency fractures?

**DOI:** 10.1007/s10195-012-0207-x

**Published:** 2012-08-01

**Authors:** Andreas Seraphim, Nawfal Al-Hadithy, Simon C. Mordecai, Shafic Al-Nammari

**Affiliations:** 1 FY1 Trauma and Orthopaedics, Lister General Hospital, Stevenage, SG1 4AB UK; 2 CT2 Orthopaedics MRCS, Lister General Hospital, Stevenage, UK; 3 SpR Orthopaedics FRCS, Lister General Hospital, Stevenage, UK

**Keywords:** Atypical, Insufficiency fracture, Subtrochanteric, Bisphosphonates

## Abstract

In recent years, several reports have suggested an association between the use of bisphosphonates and subtrochanteric insufficiency fractures. Research from animal studies and in some cases from histomorphometric data collected from patients provide evidence of a possible pathophysiological mechanism behind this phenomenon. Despite this, it has not yet been possible to confirm a causal relationship. The small number of cases, the lack of consistency in defining these atypical fractures, the absence of homogeneity between studies, and the fact that most data available are derived from retrospective observational studies, are some of the difficulties encountered in the evaluation of evidence. Despite the proven benefit of bisphosphonates at providing protection against osteoporotic fractures, caution should be used before continuing therapy for longer than 5 years.

## Introduction

Bisphosphonates have been widely used to treat and prevent osteoporosis, as they inhibit bone resorption. They have also been used in other medical conditions, including Paget’s disease [18] and glucocorticoid-induced osteoporosis [42]. Their supporting evidence is of high quality, with multiple randomised controlled trials confirming their efficacy [5, 6, 8, 24]. They act by inhibiting osteoclast activity and promoting osteoclast apoptosis [29], thereby increasing bone mineral density (BMD).

Recently, several reports have suggested a possible association between the use of bisphosphonates and nonosteoporotic fractures of the femur. Research was initially triggered by the pioneering animal study of Mashiba et al. [35] in 2000, who found reduced cortical remodelling and significant microdamage accumulation in beagles following alendronate and risedronate therapy, predisposing them to fractures. Five years later, Odvina et al. [39] reported a series of four patients with osteoporosis who whilst on bisphosphonates sustained atypical femoral fractures. Since then, there have been increasing numbers of case reports [2, 10, 13, 16, 17, 19, 22, 23, 25, 26, 30–32, 34, 38, 39, 43–46, 50, 51, 53, 54] in which atypical fracture patterns have occurred in the diaphysis or subtrochanteric region of femurs in patients on long-term bisphosphonates. The aim of this paper was to review evidence that has linked bisphosphonates with femoral insufficiency fractures.

## Diagnosis of subtrochanteric insufficiency fractures

Subtrochanteric fractures are usually rare, with an estimated 3 % of femoral fractures occurring at the subtrochanteric region [30, 37]. Being the area of the femur subjected to maximal bending stress [40], the bone remodels accordingly, rendering low-energy fractures less likely. Subtrochanteric stress fractures were traditionally seen in long-term cyclical loading injuries in bones unaccustomed to these actions—typically in new military recruits [3, 15, 28]. Diagnoses of insufficiency fractures are based on history, radiological features and bone biopsies. The fracture is frequently preceded by a history of persistent thigh pain prior to low-energy trauma. Radiographs typically reveal a transverse fracture with a medial cortical spike, with other features including cortical thickening or cortical beaking [7]. This pattern had been described as an atypical subtrochanteric fracture, and despite the lack of a clear definition, the term has been used to describe the presence of a subtrochanteric fracture with similar radiological features as a stress fracture associated with no or minor injury. In 2010, the American Society for Bone and Mineral Research published guidelines regarding the definition of an atypical fracture in an attempt to provide some homogeneity between future studies and case reports using these fractures as primary outcomes. Indeed, according to their report, an atypical fracture is defined by specific major and minor features [48]. Of note is the fact that all major features consist of specific radiological findings as well as a history of no or minimal trauma. In view of the lack of a clear distinction, the terms atypical/insufficiency fractures are used interchangeably in this review.

## Pathophysiology

It has been thought that during the prolonged time that bisphosphonates are spent in the body—half-life of 12 years—[49], osteoclastic inhibition is sequentially followed by a decrease in bone formation [12]. Bone remodelling is limited to an extent that may potentially be harmful, as prevention of this natural process eventually alters the load distribution passing through the bone. Therefore, although BMD may be preserved, the poorer quality bone and failure of aging collagen may increase susceptibility to these insufficiency fractures. Indeed, bone biopsies taken from fracture sites or the iliac crest of patients on bisphosphonates confirmed low bone turnover [38]. It has been suggested that the combination of increased bone mineralisation, together with a marked reduction of bone turnover, promote accumulation of microfractures, resulting in changes in bone mechanical behaviour [14, 55].

Chapurlat et al. [11], investigated the degree of bone turnover suppression in women who had been on long-term bisphosphonate use (mean duration 6.5 years) by obtaining transiliac bone biopsies and found significantly reduced turnover compared with those not on bisphosphonates. A similar result was found by Stepan et al. [52], who performed a cross-sectional analysis of postmenopausal women on alendronate (mean duration 5.27 years) who underwent histomorphometric biopsies.

## Case reports/case series

Since the animal study by Mashiba et al. [35], there have been increasing numbers of case reports [2, 10, 13, 16, 17, 19, 22, 23, 25, 26, 30–32, 34, 38, 39, 43–46, 50, 51, 53, 54], all of which provide typical histories of low-energy fractures and some in combination with classical radiological features. Husada et al. [25] published the first report on femoral insufficiency fractures in 2005, and although a typical history and radiological features were provided, there was no mention of the duration that the patient was on alendronate prior to the fracture. In 2006, the National Osteoporosis Foundation suggested that stopping alendronate after 5 years of continuous therapy may be beneficial. Since then, several case reports have clearly taken note of this and documented longer periods of bisphosphonate use (Table 1).

**Table 1:** Summary of case reports/case series

Author	No. of patients	Age of patient	Fracture site	Other risk factors	BPN	Years of bisphosphonate preceding injury	Histomorphometrically confirmed suppressed bone turnover
Aspenberg et al. [2]	1 F	57	Subtrochanteric	RA, pred	ALN/RSN	7	Yes
Capeci and Tejwani [10]	7 F	61	Subtrochanteric/femoral shaft		ALN	8.6	No
Cheung et al. [13]	1 F	82	Femoral shaft	No risk factors	ALN	10	Yes
Das De et al. [16]	12 F	63.1	Subtrochanteric Midshaft	4× Pred	ALN	4.6	No
Demiralp et al. [17]	1 F	65	Femoral shaft	Pred	ALN	7	No
Edwards et al. [19]	1 F	60	Femoral diaphysis	Steroid use for 10 years	ALN	6	
Goddard et al. [22]	1 F	67	Femoral shaft	No risk factors	ALN	16	No
Griffing and Nallapaneni [23]	1 M	49	Femoral shaft	HIV	ALN	8	No
Husada et al. [25]	1 F	72	Femoral shaft	Osteoporosis	ALN	Not stated	No
Ing-Lorenzin et al. [26]	7 F	67.5	Subtrochanteric	Steroid and proton pump inhibitor use	5× ALN 2× Ibadronate	ALN (16 months–8 years)	No
Kwek et al. [30]	17 F	66	Subtrochanteric	1× RA	ALN	4.8	No
				1× Pred			
Lee et al. [31]	1 F	73	Femoral shaft	–	ALN	1.5	No
Lenart et al. [32]	15 F	–	Subtrochanteric/femoral diaphysis	–	ALN	5.4	No
Leung et al. [34]	9 F 1 M	78.2	Subtrochanteric/femoral diaphysis	2× Pred	ALN	3.6	No
Odvina et al. [38]	13 F	64.3	Midshaft	4× Steroids	10× ALN 3× RSN	ALN—8.5 RSN—3.3	Yes 6/13—severe suppression
Odvina et al. [39]	4 F	63	Proximal/midshaft	1× Steroids	ALN	6.5	Yes
Sayed-Noor and Sjoden [43]	1 F	72	Subtrochanteric	Anorexia	ALN	7	No
Sayeed-Noor and Sjoden [44]	2 F	66.5	Midshaft Subtrochanteric	–	ALN	10	No
Schilcher and Aspenberg [45]	5	75	Femoral shaft	1× RA and steroid	ALN	5.8	No
Schneider [46]	1 F	59	Spiral mid-shaft	HRT	ALN	7	No
Somford et al. [50]	1 F	76	Bilateral femoral shaft	RA Pred	ALN	8	Yes
Somford et al. [51]	3 F	73.3	Subtrochanteric	3× RA, pred	ALN	9.3	No
Visekruna et al. [53]	3 F	62.7	Subtrochanteric/femoral shaft	3× Pred (11.7 years)	ALN	8.3	Yes—reduced osteoids
Wang et al. [54]	7 F 1 M	72	Subtrochanteric	5× Steroids	ALN	5.9	No

*F* female, *M* male, *RA* rheumatoid arthritis, *Pred* prednisolone, *BPN* bisphosphonate, *ALN* alendronate, *RSN* risedrontate, *HRT* hormone replacement therapy

Odvina et al. [39] published a case series of 4 patients who had subtrochanteric fractures whilst on alendronate for a mean of 6.5 years. In all cases there were normal biochemical markers of bone turnover on haematological testing. However, in three of the four patients, bone histomorphometry confirmed decreased bone turnover, and the authors estimated bone formation rate to be 100 times less than a normal postmenopausal woman. They also noted that there were minimal signs of callus formation whilst on alendronate, and in certain cases, fracture healing only occurred once alendronate therapy had stopped.

Only a few other cases [2, 13, 38, 51, 53] presented histomorphometric data collected via bone biopsies. Certain reports [38, 51, 53] demonstrated severely suppressed bone turnover following prolonged use of bisphosphonates, as histological samples showed reduced or absent tetracycline labelling and reduction in osteoclastic and osteoblastic surfaces. In other cases [2, 13], biopsy samples showed normal bone turnover and no evidence of hypermineralisation or microcrack accumulation, arguing against a potential pathophysiological mechanism. Most of these reports had substantial confounding factors, including inconsistent or poorly documented duration of bisphosphonate use, concurrent use of other medications that are known to affect bone physiology and comorbidities. Evidently, these case reports did not provide any scientific evidence of causality; however, they did highlight the need for further investigations on this topic.

## Retrospective studies

Lenart et al. [33], performed a small case–control study of 41 subtrochanteric or femoral shaft fracture, comparing them with patients sustaining intertrochanteric/femoral neck fractures. Among the 41 cases, 15 (37 %) were on bisphosphonates compared with nine of 82 (11 %) controls [odds ratio (OR) 4.4; confidence interval (CI) 1.8–11.4; *p* = 0.002]. Furthermore, their study showed that the characteristic radiological pattern associated with insufficiency fractures was highly associated with the use of bisphosphonates (OR 15.3; CI 3.1–76.9; *p* < 0.001). However, it is important to note that pretreatment radiographs were not available for comparison, raising the possibility that these radiological differences could be the result of anatomical variations between patients. Interestingly, this problem was addressed by Issac et al. [27], who examined 100 patients with low-energy femoral shaft fractures before and after bisphosphonates became available. The study compared the radiographs of 21 patients with this type of fracture before the availability of bisphosphonates (period 1995–1997) and compared them with 79 patients presenting with the same fracture over the period 2007–2009. Interestingly, none of the patients from the 1995 to 1997 period had the characteristic radiological features of insufficiency, whereas 41 patients from the bisphosphonate group had these features.

One of the largest studies was carried out by Park-Wyllie et al. [41], who performed a population-based observational study of women >68 years who were started on a bisphosphonate orally between 2002 and 2008. Over the 7-year period, 205,466 women were started on bisphosphonates, with 716 women sustaining a subtrochanteric/femoral shaft fracture (411 and 305 women, respectively). They suggested that long-term bisphosphonate treatment (>5 years) was associated with higher risk of subtrochanteric/femoral shaft fracture compared with transient use (<100 days) of bisphosphonates (adjusted OR 2.74; 95 % CI 1.25–6.02). Interestingly, shorter durations (>100 days–5 years) were not shown to be associated with an increased risk. The authors were also able to assess the validity of their study design by investigating the effect of long-term bisphosphonate use on femoral neck and intertrochanteric fractures. Indeed, the study confirmed the well-documented effect of bisphosphonates on these types of fractures, as it demonstrated their protective effect (adjusted OR 0.76; 95 %CI 0.63–0.93). Despite the statistical significance achieved in the study, it is worth emphasising the small absolute risk of subtrochanteric/femoral shaft fractures detected, as among 52,595 women on a minimum of 5 years on bisphosphonates, a fracture was seen in only 188 women. As seen in most studies investigating the role of bisphosphonates and atypical fractures, that study did not use radiological features of insufficiency or atypia for identifying cases. The study outcome was reached by confirming the absence of trauma and radiological identification of the fracture site.

Another retrospective review, by Neviaser et al. [36], reported 70 low-energy femoral shaft/subtrochanteric fractures. Twenty-five patients were taking bisphosphonates; however, the duration of bisphosphonate use was only collected for 16 patients (6.2 years). Three independent, blinded orthopaedic surgeons where asked to review the radiographs of these 70 cases and identify those with characteristic radiological features suggestive of insufficiency fractures (simple, transverse, or short-oblique pattern in areas of cortical thickening with a unicortical beak). The authors concluded that the characteristic fracture pattern was much more common in patients on bisphosphonates (76 %) compared with only 2 % not on bisphosphonates. They also found that this pattern was 98 % specific to alendronate users. Adding further evidence to their conclusion was their finding that patients taking alendronate for longer (6.9 years) were more likely to have the classical radiological features than those on a shorter duration (2.5 years). Apart from the study being retrospective, other limitations included limited patient numbers and that confounding factors such as glucocorticoid use and other patient comorbidities were not taken into account.

Giusti et al. [21] performed a systematic review of 141 atypical fractures that occurred in patients taking bisphosphonates, with a mean age of 68.8 and duration of 5.95 years bisphosphonates therapy. Their work suggested there was no association between bisphosphonate use and atypical fractures, as patients who took bisphosphonates for <5 years, were more likely to have femoral shaft fractures compared with those taking it for >5 years. Nevertheless, the quality of this study emphasised the actual quality of most reports, as vital information such as patient adherence to medication, comorbidities, other medications, dose of bisphosphonates and bone turnover markers were often not recorded. More importantly, the authors reported that in >50 % of case reports, important data was not adequately reported or was completely missing.

Evidence against an association between bisphosphonates and atypical subtrochanteric fractures also comes from the work of Abrahamsen et al. [1]. They performed a register-based matched cohort analysis and found that the ratio of typical osteoporotic fractures and atypical subtrochanteric fractures was identical in alendronate-treated fracture patients and their matched untreated controls. Nevertheless, the main limitation of their study was that they were unable to truly identify the number of atypical subtrochanteric fractures. Fracture diagnosis was based on the International Classification of Diseases (ICD)-10 coding used in the health register, and it was therefore not possible to differentiate between high- and low-energy fractures, which are one of the key diagnostic criteria. Furthermore, because of the study design, it was not possible to perform any radiological assessment of the fracture sites and thus no criteria for defining atypical/insufficiency fractures were used.

## Randomized controlled trials

Bone et al. [9] performed a randomised, double-blind, placebo controlled trial of the effects of alendronate on bone density and histomorphometry in 425 postmenopausal women. They found that alendonate alone or in combination with oestrogen did not affect histomorphometry and concluded that alendronate produced favourable effects on BMD. Despite the strength of the study design, there were certain limitations. Firstly, patients were only on alendronate therapy for a maximum of 2 years, and as previously suggested, this might not be long enough. Secondly, of the 425 patients, only 98 underwent bone biopsies, and there was no mention of the selection process and reasons for selection.

The largest study was by Black et al. [7], who performed secondary analyses of three large randomised controlled trials of bisphosphonates. Two trials were based on alendronate therapy: the Fracture Intervention Trial (FIT) [5] and FIT Long-Term Extension (FLEX) [47]; a third trial was based on zoledronic acid: the Health Outcomes and Reduced Incidence with Zoledronic Acid Once Yearly—Pivotal Fracture Trial (HORIZON–PFT) [20]. In the FIT trial, 6,549 women over the age of 65 years were randomised to either receive alendronate or placebo for 3–4.5 years. The FLEX continued follow-up in 1,099 of the 6,549 women in the FIT trial over the subsequent 10 years. In the HORIZON–PFT trial, 7,736 women were randomised to either receive zoledronate or placebo over 3 years. Between the three trials, 284 hip fractures were found, and only 12 were subtrochanteric or midshaft femoral fractures. Due to the low incidence of these insufficiency fractures, Black et al. concluded that there was no increased risk associated with prolonged bisphosphonate therapy. Despite the large numbers of patients on alendronate therapy, the authors themselves eluded to the fact that their study was underpowered for definitive conclusions. Further weaknesses include the short duration of bisphosphonate therapy in most cases. Only 1,000 of 15,384 patients took bisphosphonates for >4 years. Another limitation in the process was the lack of radiographs, and therefore the inability to assess for signs of atypia. The diagnosis of insufficiency fracture was again based on fracture site as per radiological report and history of a low-energy fracture.

The same limitations are also encountered in another report by Bilezikian et al. [4], who reported the incidence of these atypical fractures in a randomized, placebo-controlled, phase III trial of the use of risedronate in postmenopausal women with osteoporosis. The trial, which enrolled >15,000 patients, found no causal relationship between risendronate use and this type of fracture. Nevertheless, the mean duration of bisphosphonate use was only 1.9 years, an observation that, as explained previously, could potentially alter the outcome of the study.

## Conclusion

Based on the published literature, femoral insufficiency fractures appear to be a different pathology compared with standard osteoporotic fractures, and the atypical features of the fracture are rarely seen without long-term bisphosphonate therapy. Even so, evidence from other studies suggests that these fractures are, in reality, part of the natural history of osteoporosis. Due to the rarity of these fractures, however, there are a limited number of cases published and very few adequately powered studies. Almost all evidence is derived from retrospective observational studies, and in the majority of cases, data are either incomplete or complicated by several confounding factors. Evidently, there is some evidence collected from randomized controlled trials; however, it is worth emphasising the fact that these trials were not designed to investigate this association and are therefore weakened by the lack of important data.

Nevertheless, evidence from the literature confirms the existence of a type of fracture that has specific radiological features and could potentially be associated with the use of bisphosphonates. It is therefore important to consider that even if a causal relationship is shown, it would still be extremely difficult to question the clinical use of bisphosphonates. The risk/benefit ratio of bisphosphonates has been documented in multiple good-quality studies. This ratio is unlikely to be significantly shifted, as both the incidence of these fractures—31 per 10,000 patient years in women receiving alendronate—[1], and the potential absolute risk associated with the use of bisphosphonates seems to be relatively small. Even so, caution should be used before continuing therapy for >5 years, and prodromal symptoms should be taken into consideration. More well-designed studies are needed to establish the presence of a causal relationship.
